# Recent Applications of Diversity-Oriented Synthesis Toward Novel, 3-Dimensional Fragment Collections

**DOI:** 10.3389/fchem.2018.00460

**Published:** 2018-10-16

**Authors:** Sarah L. Kidd, Thomas J. Osberger, Natalia Mateu, Hannah F. Sore, David R. Spring

**Affiliations:** Department of Chemistry, University of Cambridge, Cambridge, United Kingdom

**Keywords:** fragment-based drug discovery, diversity-oriented synthesis, medicinal chemistry, organic synthesis, compound collections

## Abstract

Fragment-based drug discovery (FBDD) is a well-established approach for the discovery of novel medicines, illustrated by the approval of two FBBD-derived drugs. This methodology is based on the utilization of small “fragment” molecules (<300 Da) as starting points for drug discovery and optimization. Organic synthesis has been identified as a significant obstacle in FBDD, however, in particular owing to the lack of novel 3-dimensional (3D) fragment collections that feature useful synthetic vectors for modification of hit compounds. Diversity-oriented synthesis (DOS) is a synthetic strategy that aims to efficiently produce compound collections with high levels of structural diversity and three-dimensionality and is therefore well-suited for the construction of novel fragment collections. This Mini-Review highlights recent studies at the intersection of DOS and FBDD aiming to produce novel libraries of diverse, polycyclic, fragment-like compounds, and their application in fragment-based screening projects.

## Introduction

Within the biomedical community there remains a pressing need for new molecules to seed early stage drug discovery programs. Diversity-oriented synthesis (DOS) emerged in the early 2000s in response to this challenge, a strategy which involves the efficient and deliberate construction of multiple scaffolds in a divergent manner (Lee et al., 2000; Schreiber, 2000; Spring, 2003; Burke and Schreiber, 2004). Nowadays, applications of this methodology span much of the spectrum of chemical space with examples describing the synthesis of fragment(Hung et al., 2011), small molecule (Wyatt et al., 2008; Lenci et al., 2015; Caputo et al., 2017), peptide (Kotha et al., 2013; Contreras-Cruz et al., 2017; Zhang et al., 2017) and macrocyclic (Isidro-Llobet et al., 2011; Kopp et al., 2012; Beckmann et al., 2013; Dow et al., 2017) collections all abundant within the literature. Furthermore, as the field of DOS has evolved, research themes have focused on addressing key calls from within the drug discovery community, namely the deficiencies within compound screening libraries (Lipkus et al., 2008; Dow et al., 2012), the identification of new bioactive molecules against challenging biological targets (Stanton et al., 2009; Kato et al., 2016; Kim et al., 2016) and populating underexplored areas of chemical space with novel structural entities (Thomas et al., 2008; Morton et al., 2009; Pizzirani et al., 2010). Until recently, however, the majority of DOS successes have been achieved in high-throughput screening (HTS) contexts (Chou et al., 2011; Laraia et al., 2014; Aldrich et al., 2015; Kuo et al., 2015).

Recent applications of DOS, however, exemplify how this methodology can be utilized to address significant challenges currently faced within the field of fragment-based drug discovery (FBDD). FBDD is now a widely adopted technique across both industry and academia, with two marketed drugs having emerged from this methodology [vemurafenib (Bollag et al., 2012), venetoclax (Souers et al., 2013)] and dozens of clinical candidates (Erlanson et al., 2016). This process involves the screening of small “fragment” molecule libraries (<300 Da) to identify efficient, but none the less weakly binding molecules, which are in turn subsequently elaborated to generate potent lead compounds (Erlanson and Jahnke, 2016). “Rule of three” guidelines are commonly employed within FBDD and library construction, relating to a molecular weight <300 Da, the number of hydrogen and acceptors/donors ≤3 and a cLogP ≤3 (Congreve et al., 2003). Importantly, due to the additional physicochemical constraints imposed on these screening libraries compared to traditional HTS approaches, it is broadly accepted that this method allows far more efficient sampling of chemical space, since there are far fewer possible fragment-sized molecules (Murray and Rees, 2009; Hall et al., 2014).

Despite significant advances in the foundational technologies of FBDD which have aided its implementation, reports associated with the synthetic intractability of hit fragments support the view that organic synthesis may be a rate-limiting step in the FBDD cycle, and in fact across drug discovery as a whole (Murray and Rees, 2016; Blakemore et al., 2018). In a similar vein to traditional drug discovery, deficiencies in commercially available fragment screening collections have been noted, in particular relating to the overrepresentation of sp^2^-rich flat molecules (Hajduk et al., 2011; Hung et al., 2011) that feature limited numbers of synthetic handles for fragment elaboration. This latter feature is especially important within FBDD since this process relies on the merging, growth or linkage of small fragment molecules to develop initial weak hits (typically μM or mM range) hits into potent lead compounds. Without these vital functional handles, this process is significantly more time consuming, requiring the development of new synthetic routes to modify relatively simple fragment scaffolds. Furthermore, the incompatibility of many existing synthetic methodologies with amines, heterocycles, and unprotected polar functionalities limits their utilization. Consequently, there is a need for new strategies and technologies that enable non-traditional disconnections, late-stage functionalization as well as the incorporation of 3D elements into drug-like scaffolds.

Thus, appeals from within scientific community have been made for the development of novel and flexible synthetic methodologies that enable access to new fragments and their derivatives, including those with increased 3-dimensionality and heterocyclic architecture (Keseru et al., 2016; Murray and Rees, 2016). Despite the debates within the literature on the requirements of 3D character within fragment libraries, population of these underrepresented areas can be considered to complement existing flatter libraries, whilst providing access to alternative growth vectors, and therefore remains an important avenue of research (Morley et al., 2013; Fuller et al., 2016). From the perspective of library construction, the 3D character of the resulting libraries is commonly judged by the number of chiral centers and the fraction of sp^3^ carbons (Fsp^3^) within a molecule (Lovering et al., 2009), in addition to visual representations of the molecular shape space distribution using principal moment of inertia (PMI) analysis (Sauer and Schwarz, 2003; Kopp et al., 2012).

With a growing demand for novel heterocycles and 3D-shaped molecules for use within FBDD campaigns, many studies centering on the synthesis of 3D fragments around single heterocycles have been reported, for example using C-H activation methodologies (Davis et al., 2015; Palmer et al., 2016; Antermite et al., 2018). This mini-review aims to highlight the suitability of DOS approaches for addressing these challenges through the production of multiple scaffolds with a broader coverage of chemical space. One important feature of this strategy is the utilization of highly efficient and modular synthetic routes, commonly in the form of a build/couple/pair (B/C/P) algorithm (Nielsen and Schreiber, 2008). This involves (1) *the build phase*—construction of common starting materials, (2) *the couple phase*—intermolecular coupling of the building blocks with readily synthesized or commercial materials to form reactive intermediates and (3) *the pair phase*—intramolecular reaction or cyclisation of these precursors to afford distinct scaffolds. Thus, the flexibility of these strategies often results in methodologies that can provide efficient access to analogs of a desired scaffold. Herein, we discuss recent applications of the DOS strategy to the construction of novel and diverse 3D fragment collections and their applications in FBDD.

## The application of DOS to access novel fragments with multiple growth vectors

**Figure 1:**
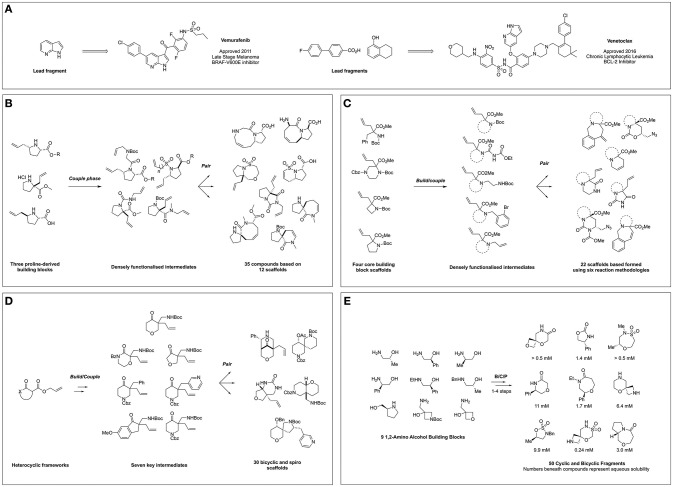
FBDD-derived drugs and diversity-oriented synthesis of novel fragment libraries. **(A)** Clinically approved drugs derived from FBDD and their lead fragments. **(B)** Hung et al. (2011) Report on DOS of fragment-like molecules. **(C)** Foley et al. (2015) Lead-like modular route to novel scaffolds. **(D)** Mayol-Llinàs et al. (2017) Lead-like scaffold DOS for CNS and FBDD. **(E)** Haftchenary et al. (2016) DOS of aqueous soluble fragments from 1,2-amino alcohols.

The first publication conceptually merging DOS and FBDD appeared in 2011 in which Hung et al. (2011) described the application of DOS for the generation of a 3D fragment collection utilizing allyl proline-based precursors as the basis for library design. The researchers exploited three proline-derived building blocks in a B/C/P sequence to facilitate the formation of a series of fused and spiro bicyclic compounds (Figure 1B). This was achieved through installation of a second olefin *via N*-substitution using a variety of linker types, furnishing distinct linear precursors. Subsequently subjecting these intermediates to various intramolecular cyclizations such as ring closing metathesis (RCM) and oxo-Michael reactions, yielded 20 compounds based on 12 frameworks. Furthermore, due to the modular nature of this approach a complete matrix of stereoisomers of the 5–6, 5–7, 5–8, and 5–9 bicyclic frameworks could be constructed. Finally, the scaffolds were derivatized in a post-pair phase manner through functional group interconversion or olefin reduction to increase the diversity and the saturation, affording a total of 35 fragments.

Importantly, polar functional handles were installed throughout the library, enabling potential fragment growth from different vectors during hit-to-lead efforts. The applicability of the resultant library to FBDD was demonstrated *via* chemoinformatic analysis, which highlighted rule of three compliance whilst principle moment of inertia (PMI) plots suggested a broad coverage of 3D molecular shape space.

Amino acid-derived reagents represent valuable building blocks for use within DOS methodologies owing to their polar and chiral nature, and their exploitation within these techniques has become more prevalent within the field. Work by Foley et al. (2015) described the application of four α,α-amino acid derived building blocks to generate a library of diverse bicyclic and tricyclic fragments (Foley et al., 2015). Through variation in the building block structure and the nature of the pair-phase cyclisation the researchers constructed 22 different heterocyclic scaffolds in a synthetically efficient manner (Figure 1C). Firstly, five different nitrogen substituents were installed on the four amino acid building blocks: a *tert*-butyl carbamate, an acyl urea, a 1,2-diamine, a *o*-bromobenzylamine or a second allyl olefin. In turn, pair phase reactions were then explored through reactivity of these functionalities with either the preinstalled ester or allyl moieties. This included iodine-mediated cyclisation followed by azide addition and reactivity of the electrophillic ester moiety with *N*-based nucleophiles. Finally, ring closure *via* either Pd-mediated Heck reaction or Ru-mediated metathesis afforded further tri- and bicyclic fragments. The final collection of 22 scaffolds featured biologically relevant moieties such as ureas, hydantoins, and lactams, in addition to multiple functional synthetic handles. Subsequent virtual enumeration led to a library of 1,110 compounds that were predicted to possess lead-like properties and with considerable 3D character, (average Fsp^3^ = 0.57) and several examples meeting the criteria for FBDD.

In a similar vein, Mayol-Llinàs and co-workers also explored the use of cyclic α-allyl quaternary ketones in a divergent and modular synthetic process to generate a library of 30 structurally distinct scaffolds featuring spiro, fused and bridged architectures (Figure 1D) (Mayol-Llinàs et al., 2017). Instead of amino acid-based precursors, Tsuji-type decarboxylative allylation was utilized to generate seven quaternary allylated building blocks. One example was selected for pilot studies, during which a variety of transformations were applied in a reagent-based approach to yield 12 different scaffolds through exploitation of four key reactive moieties within the intermediate. This included an intramolecular Mannich reaction, a sequence of hydroboration-oxidation followed by either reduction or sulfonylation and then cyclisation, base-mediated cyclisation and Pd-catalyzed aminoarylation. Then, the remaining six precursors were subjected to the most promising conditions, yielding an additional 18 scaffolds. Virtual library enumeration was also conducted using six synthetic transformations, including reductive amination, urea formation and sulfonylation using 98 medicinal chemistry relevant capping groups. Multiparameter optimization analysis (Wager et al., 2010) was used to assess the amenability of this work to a CNS-based drug discovery context. In addition, it was noted the resulting library possessed lead-like properties (Doveston et al., 2014) and that many of the compounds and derivatives would be applicable to a FBDD setting.

A recent report from Haftchenary at the Broad Institute detailed the synthesis of a fragment collection based on chiral 1,2-amino alcohols (Figure 1E) (Haftchenary et al., 2016). Beginning from a library of nine readily available amino alcohols, a range of 5-, 6-, and 7-membered scaffolds were synthesized in 1–4 steps using established synthetic procedures. The resulting fragment collection included medicinally important heterocycles such as oxazolidinones, morpholinones, and sulfamidate and sultam-based rings, along with fused and spiro-bicyclic compounds. Importantly for a screening context, the aqueous solubility of each of the 50 final fragments was measured, with values ranging from 0.085 to >15 mM, within the range for many fragment screening techniques.

**Figure 2:**
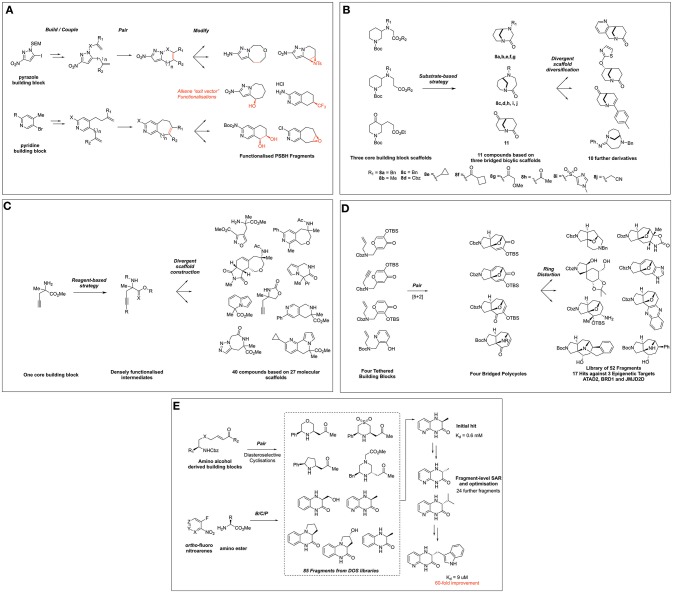
Fragment collections derived from DOS and their application to FBDD. **(A)** Twigg et al. (2016) DOS of partially saturated bicyclic heteroaromatic fragments. **(B)** Hassan et al. (2018) DOS fragment library based on twisted amides. **(C)** Mateu et al. (2018) DOS fragment library based on α,α-disubstituted amino esters. **(D)** Foley et al. (2017) synthesis of scaffolds distantly related to natural products. **(E)** Wang et al. (2016) DOS fragment evolution strategy against GSK3.

Further to the goal of populating fragment space with sp^3^-enriched compound collections that possess favorable fragment-like properties and synthetic exit vectors, Spring and coworkers disclosed a DOS-related approach to the synthesis of partially saturated bicyclic heteraromatic (PSBH) molecules (Figure 2A) (Twigg et al., 2016). The synthetic route centered on the functionalization of pyrazole and pyridine-based building blocks featuring possessing amino-, or nitro groups, which were incorporated as potential solubilizing moieties; alternatively, a chloro substituent was incorporated as a hydrophobic element. The build and couple stages comprised of Suzuki cross coupling and alkylations to install various alkene functionalities, which were paired using RCM to afford bicyclic scaffolds, each featuring a positionally defined endocyclic alkene vector for further functionalization.

The endocyclic alkene was then modified in a post-pairing event to exemplify its utility as a synthetic growth vector in these fragments. A variety of alkene transformations, including dihalogenation, epoxidation, aziridination, cyclopropanation, halohydrin formation, and hydroboration-oxidation, were performed to yield a range of further functionalized scaffolds. The resulting library of compounds was then subjected to analysis of its physicochemical properties, which compared favorably to commercial screening libraries in relation to key properties such as number of chiral centers (0.88 vs. 0.27 or 0.18) and fraction aromatic (0.43 vs. 0.42 and 0.52), while maintaining rule of three compliance.

Hassan et al. recently disclosed an interesting example via the exploitation of twisted bicyclic amide compounds for the generation of a 3D fragment screening library (Hassan et al., 2018). In this work five 3-(ω-carboxylate)-substituted piperidine starting materials were manipulated to produce a 22-member polycyclic library (Figure 2B). Using substrate-based DOS methodology, five analogous starting materials based on three common structures were constructed and *via* Bu_2_SnO-mediated cyclisation these were transformed to afford bicyclo[4.3.1]decane and bicyclo[3.3.1]nonane scaffolds in moderate yield. The generality of this methodology was exemplified through the synthesis of six further compounds through modification of the *N*-substituent the bicyclic ring systems.

In turn, these three key scaffolds were ultimately then divergently modified through manipulation of either the ketone or amide functionalities to generate a further nine compounds. The ketone moiety within the bicyclo[3.3.1]nonane was first modified by the use of either gold- or palladium-mediated reactions to afford tetra- or tricyclic heteroaromoatic fused motifs. Alternatively, this moiety could also be reduced and a variety of heteroaromatics or alkyl moiety installed *via* either S_N_Ar or alkylation conditions in a diastereoselective fashion. Finally, the twisted amide within these scaffolds could also be manipulated to form either a chloroenamine intermediate, followed by Suzuki-coupling to install an aryl substituent or simply by amidine formation. The resultant library was shown to possess fragment lead-like properties with a high Fsp^3^ (0.63) and generally 17 or fewer heavy atoms and a clogP < 2.5. Furthermore, PMI analysis of the shape distribution suggested the library possessed significant 3D character to complement existing fragment collections for screening purposes.

The most recent and final example of synthetic efforts within this field by Mateu et al. (2018) report the use of α,α-disubstituted amino esters for the DOS of fragments incorporating a *N*-substituted quaternary carbon, an important and underrepresented motif within screening collections (Figure 2C). Using a single building block, 40 structurally diverse molecules based on 27 molecular frameworks were constructed in a synthetically efficient manner using an average of only three synthetic steps to access the entire library. This involved exploiting the three reactive handles within the building block in different combinations and utilizing a broad range of chemistries such as [2+2+2] cyclotrimiserizations, Au-, Ru-, and Cu- mediated cyclizations and regioselective click chemistry to afford mono-, bi-, and tri-fused heterocycles featuring this important motif. Importantly, the authors also demonstrated the versatility of this synthetic methodology through the synthesis of an alternative quaternary *R*-substituent and the asymmetric synthesis of one library member.

Subsequent computational assessment of the resulting library *via* PMI analysis revealed a broad distribution of molecular shape space, in addition to favorable comparisons to a commercially available fragment collection in terms of 3-dimensional shape space coverage. Additionally, the mean values of the physicochemical properties of the library demonstrated the compatibility of the library for fragment screening, falling within the Rule of three guidelines, whilst exhibiting more favorable properties when again compared to existing commercial libraries. The authors note promising hits identified by X-ray fragment screening at the XChem screening facility, against proteins from three distinct families (a hydrolase, a TGF β growth factor, and a peptidase).

## Demonstration of DOS methodologies for the identification of novel binders for challenging biological targets

In addition to populating new areas of fragment chemical space, DOS-derived fragment libraries can play a significant role in the identification of novel binders to seed future FBDD programs. Recent work by Foley et al. (2017) demonstrated the application of DOS-derived fragment libraries in the identification of novel hits against three epigenetic proteins from two distinct mechanistic classes (ATAD2, BRD1, and JMJD2D), *via* X-ray crystallographic screening methods. The researchers took inspiration from natural product frameworks, utilizing intramolecular [5+2] cyclizations to forge bridged structures incorporating natural product-related heteroaromatic frameworks (Figure 2D). Ring distortion reactions on these four initial structures using either expansion, cleavage, annulation, or substitution methodologies, were performed to divergently modify the precursors, ultimately affording a library of 52 fragments based on 23 different scaffolds with bridged architectures and a high sp^3^ content. Interestingly, when this library was screened against the three epigenetic targets *via* high-throughput X-ray crystallography methods, 17 hits were identified against the three proteins, including those binding in novel regions of the proteins to those described previously. Moreover, comparisons could be drawn between the natural product-like fragment library and that obtained from commercial sources, whereby a significantly higher hit rate against ATAD2 was observed with the 3D fragments synthesized where seven hits were identified from a 52-member library vs. the commercially available fragment library where nine hits were identified from a 700-member library. Although the authors did not report any biophysical data for the fragments, the identification of novel X-ray hits from these efforts demonstrate the promise of a merged DOS-FBDD approach.

Finally, Young and co-workers recently demonstrated the successful use of the DOS strategy to optimize fragments against the serine/theronine kinase GSK3β (Wang et al., 2016), which is overexpressed in cancer and Alzheimer's disease (Luo, 2009; Hernandez et al., 2012). To initiate the investigation, a set of 86 fragments was compiled from DOS libraries constructed *via* three distinct B/C/P pathways (Figure 2E). The first DOS fragment library utilized allylproline building blocks and has been previously discussed in this review (Figure 1A). The second DOS library coupled enones with amino alcohol and related building blocks. The final scaffolds were accessed *via* catalytic, diastereoselective aza-Michael additions to afford stereochemically diverse disubstituted heterocycles. The third DOS library incorporated into this study was generated from *ortho*-nitrofluoro arenes and α-amino ester building blocks. Intermolecular coupling products were obtained *via* S_N_Ar, and pairing products were accessed by reduction of the nitro group followed by spontaneous cyclisation onto the ester functionality. This modular approach yielded a small collection of enantiomerically enriched bicyclic piperazinone compounds.

Using this fragment collection, screening against GSK3β was performed using differential scanning fluorimetry (DSF) to detect fragment binding. Initial results identified a benzopiperizinone-library member to exhibit good thermal stabilization and subsequent assays showed 46% inhibition of GSK3β at 1 mM concentration. A library of derivatives based on this initial hit were then synthezised using the modular and rapid DOS chemistry initially developed. Thus, the single enantiomer variants and other derivatives could easily be constructed to generate structure-activity relationships (SAR). Preliminary fragment-level SAR indicated the *(R)*-enantiomer of the chiral center to be more potent, and further studies identified the substituent at this site as an important potential growth vector. Fragment growth by incorporating large aryl groups into the scaffold *via* the same B/C/P pathway yielded the lead compound, with a large indolyl unit connected to the core heterocycle. This fragment exhibited a K_d_ = 9 μM, a 60-fold improvement over the initial fragment hit. Ultimately an X-ray crystal structure of the lead compound with GSK3β was obtained, revealing it binds in the ATP pocket of this kinase.

This study demonstrated the successful implementation of a DOS-based FBDD workflow to evolve fragments against an important kinase target. Key to the success of this project was the utility of the DOS concept as a tool to generate skeletally and stereochemically diverse initial libraries, and later as an efficient, modular route to analogs for SAR and fragment growth.

## Future perspectives

The studies discussed herein have demonstrated the utility of DOS as an effective approach for populating new areas of fragment space, in areas largely complementary to existing fragment collections. In each case, the resulting libraries featured high structural and shape diversity, increased 3D character and exemplified synthetic vectors for fragment growth. The latter two examples discussed detail applications of these libraries for the identification of novel fragment binders and inhibitors against challenging protein targets, ultimately demonstrating the utility of DOS within drug discovery efforts.

It is worth noting the increasing application of computational virtual library enumeration, an element of which has featured in several of the publications discussed. It is envisioned that these methodologies will only increase in their utility when coupled to *in silico*-based screening techniques to guide library design and prioritization of synthesis. Moreover, a focus on applications of newly developed methodologies to DOS, for example C-H activation, and site selective late-stage modifications of complex scaffolds would enable population of underexplored areas of chemical space and further derivatization of the resulting scaffolds. Finally, an outstanding requirement within this field is the establishment of new translational collaborations between academic and industrial groups to enable the routine screening of the novel libraries.

## Author contributions

SLK and TJO conceived and wrote the manuscript. All other authors (NM, HFS and DRS) provided comments and discussion on the manuscript to aid its preparation.

### Conflict of interest statement

The authors declare that the research was conducted in the absence of any commercial or financial relationships that could be construed as a potential conflict of interest.
